# Personalized breast cancer screening strategies: A systematic review and quality assessment

**DOI:** 10.1371/journal.pone.0226352

**Published:** 2019-12-16

**Authors:** Marta Román, Maria Sala, Laia Domingo, Margarita Posso, Javier Louro, Xavier Castells

**Affiliations:** 1 Department of Epidemiology and Evaluation, IMIM (Hospital del Mar Medical Research Institute), Barcelona, Spain; 2 Network on Health Services in Chronic Diseases (REDISSEC), Spain; Weill Cornell Medical College in Qatar, QATAR

## Abstract

**Background:**

The effectiveness of breast cancer screening is still under debate. Our objective was to systematically review studies assessing personalized breast cancer screening strategies based on women’s individual risk and to conduct a risk of bias assessment.

**Methods:**

We followed the standard methods of The Cochrane Collaboration and PRISMA declaration and searched the MEDLINE, EMBASE and Clinical Trials databases for studies published in English. The quality of the studies was assessed using the ISPOR-AMCP-NPC Questionnaire and The Cochrane Risk of Bias Tool. Two independent reviewers screened full texts and evaluated the risk of bias.

**Results:**

Out of the 1533 initially retrieved citations, we included 13 studies. Three studies were randomized controlled trials, while nine were mathematical modeling studies, and one was an observational pilot study. The trials are in the recruitment phase and have not yet reported their results. All three trials used breast density and age to define risk groups, and two of them included family history, previous biopsies, and genetic information. Among the mathematical modeling studies, the main risk factors used to define risk groups were breast density, age, family history, and previous biopsies. Six studies used genetic information to define risk groups. The most common outcome measures were the gain in quality-adjusted life years (QALY), absolute costs, and incremental cost-effectiveness ratio (ICER), while the main outcome in the observational study was the detection rate. In all models, personalized screening strategies were shown to be effective. The randomized trials were of good quality. The modeling studies showed moderate risk of bias but there was wide variability across studies. The observational study showed a low risk of bias but its utility was moderate due to its pilot design and its relatively small scale.

**Conclusions:**

There is some evidence of the effectiveness of screening personalization in terms of QUALYs and ICER from the modeling studies and the observational study. However, evidence is lacking on feasibility and acceptance by the target population.

**Review registration:**

PROSPERO: CRD42018110483

## Introduction

There is general agreement that mammography screening reduces mortality from breast cancer by 20% in invited women [1]. At present, age is the only factor used to define the target population for breast cancer screening in average-risk women. Although recommendations differ on the age range for screening and the screening interval, most countries in Europe offer biennial mammography to women aged 50 to 69 years [2], while in the US the recommendations vary with either annual or biennial screening recommendations from ages 45 to 74 years old [3,4].

There is also evidence that mammography screening may cause harms, the most widely discussed being the percentage of overdiagnosis, which is estimated by observational studies to vary between 0% and 36% of breast cancers diagnosed during the screening period [1]. Other harms of screening include the presence of interval breast cancers, representing one in every four cancers in the screening population [5], and false-positive screening results, affecting one in every five women during the course of 10 biennial screening exams [6–8].

The current standard of care for breast cancer screening offers women in the target population a homogenous strategy based solely on age, although recommendations may differ for specific subgroups of high-risk women [9–12]. In response to the need to improve the risk-benefit balance of mammography screening, some authors have proposed modifying the current uniform strategy toward more personalized screening strategies, in which women would be invited to screening according to their risk of developing breast cancer and an individually-defined plan [13].

Several studies have evaluated personalized screening strategies based on individual risk of breast cancer using different methodologies and outcomes. However, syntheses conducted in this area highlight considerable challenges, such as the difficulties of systematic evaluation of the quality of evidence of individualized screening strategies. Clearly, the existence of evidence does not mean that it will be adopted into practice. Therefore, general recommendations on whether personalized breast cancer screening strategies should be implemented should be based on high-quality evidence.

Our aim was to systematically review studies assessing personalized screening strategies based on women’s individual risk of breast cancer and to assess the quality of the evidence.

## Methods

We performed a systematic review of the literature following the standard Cochrane Collaboration methods [14] and adhering to the PRISMA (Preferred Reporting Items for Systematic Reviews and Meta-Analyses) statement reporting recommendations [15].

### PICO question

The Patient, Intervention, Comparison, Outcomes (PICO) question of this systematic review was the following: Should risk-based screening vs. the current “one-size fits all” recommendation be used in the general population of women targeted in breast cancer screening to improve the risk-benefit balance of this practice?

### Data sources and searches

We retrieved relevant literature by using a combination of controlled vocabulary and keyword search terms in the following databases: i) MEDLINE (accessed through PubMed); ii) The Cochrane Library (accessed through Wiley); iii) EMBASE (accessed through Ovid); and iv) clinical trials databases (U.S. National Library of Medicine Clinicaltrials.gov [https://clinicaltrials.gov/], International Clinical Trials Registry Platform of the World Health Organization [http://apps.who.int/trialsearch/], and the ISRCT registry [https://www.isrctn.com/]). We adapted the search algorithms to the requirements of each database and used validated filters to retrieve primary studies as needed. We reviewed the references of included studies that could potentially meet our eligibility criteria. The detailed search strategy is reported in S1 File. We searched each database from its inception up to January 2018.

### Study selection

Eligible studies were those published in English that evaluated risk-based strategies to personalize breast cancer screening. All study designs were considered potentially adequate. We excluded narrative reviews, letters to the editor, editorials, and conference communications. We excluded studies that assessed screening strategies without considering woman’s individual risk to address the proposed intervention. We also excluded studies targeted solely at the high-risk population without an intervention assessed in a comparison group of women with lower risk. These criteria ensured that we included only studies assessing personalized strategies in the general population targeted to breast cancer screening.

If the same study provided multiple publications, we selected the most detailed report on the study characteristics. Citations identified from the search were loaded into EndNote X7.7.1 for Windows (2016) to manage duplicates as well as to perform the screening based on titles and abstracts.

### Data extraction

**Fig 1:**
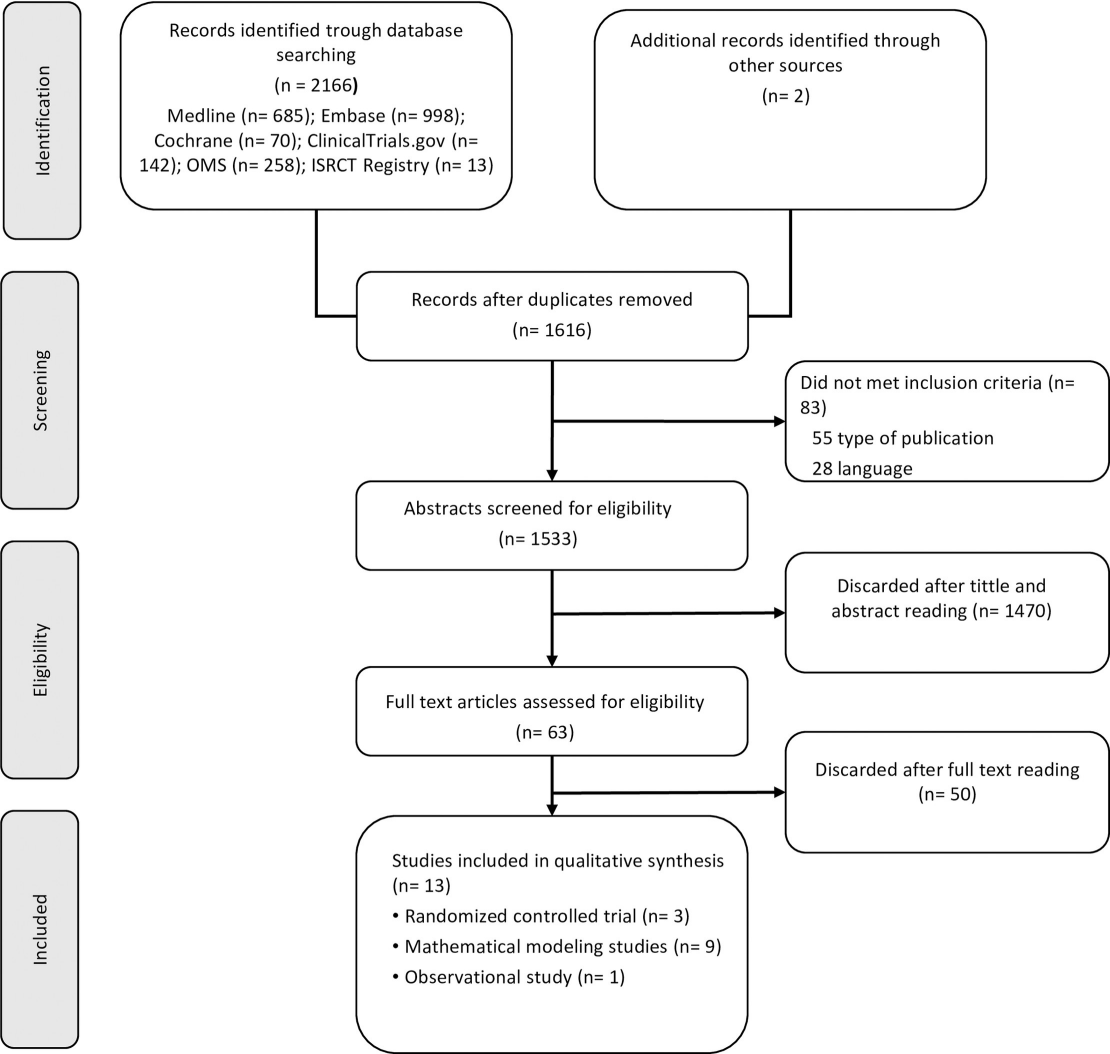
PRISMA flowchart.

A first reviewer screened the search results based on title and abstract. A second reviewer checked the quality of the screening by reviewing 20% of the references. Two reviewers independently confirmed eligibility based on the full text of the relevant articles. If there was disagreement between the reviewers, the inclusion of studies was determined by consensus. The result of this process is reported in a PRISMA flowchart (Fig 1).

We extracted the essential information from included studies. The information reported varied depending on the study design. Data extraction was conducted by one reviewer and checked by the other.

### Quality assessment

Two reviewers assessed the risk of bias independently, and the final quality assessment was based on consensus.

The risk of bias was assessed using two tools. For the mathematical modeling studies and for the observational study, we adapted the ISPOR-AMCP-NPC Questionnaire [16] to assess the relevance and credibility of each modeling study according to the following criteria: i) validation; ii) bias due to the study design; iii) limitations in data sources; iv) appropriateness of the model analysis; v) reporting bias; vi) interpretation bias; and vii) conflict of interest. The risk of bias for each domain was rated as low, high, or unclear.

To assess the risk of bias in the randomized controlled trials, we used the Cochrane Collaboration tool, specifically developed to evaluate this type of design (Cochrane Handbook for Systematic Reviews of Interventions) and available online [http://methods.cochrane.org/bias/assessing-risk-bias-included-studies] [14]. The Cochrane tool assesses the following criteria: i) selection bias (random sequence generation, and allocation concealment); ii) performance bias (blinding of participants and personnel); iii) detection bias (blinding of outcome assessment); iv) attrition bias (incomplete outcome data); v) reporting bias (selective reporting); and vi) other possible biases.

### Data synthesis and analysis

The different study designs and the heterogeneity of the outcomes reported in the studies precluded the possibility of pooling data across the studies. Therefore, a narrative synthesis was conducted. Key study characteristics and methodological quality are described in Tables 1 and 2 and are summarized in a narrative manner. The results are presented according to the study design.

**Table 1:** Characteristics of mathematical modeling studies.

Study ID	Type of modeling	Reference population	Risk factors ^1^	Comparison groups	Strategies	Outcome measures
Trentham-Dietz 2016	CISNET (microsimulation). Three different models	Women 50–74 years	Combination of mammographic density, and 4 levels of relative risk (RR: 1.0, 1.3, 2.0, 4.0) based on previously published evidence.	Reference relative risk (RR = 1) vs. RR> 1.3, RR>2.0, RR>4.0	• Annual, biennial, triennial screening age 50–74 years vs no screening • Biennial screening age 50–64 years vs biennial screening 65–74 years	Lifetime cost of breast cancer deaths, life expectancy and number of QALY, false-positive results, biopsies with a benign result, overdiagnosis, cost-effectiveness, and ratio of false-positive results among breast cancer deaths avoided by screening.
O'Mahony 2014	Cost-effectiveness microsimulation, and MISCAN (Monte Carlo microsimulation)	Women 50–70 years	Increase or decrease in breast cancer incidence in the population (continuous value)	Different screening periodicities based on breast cancer annul incidence. Taking as reference a cost-effectiveness threshold of €20,000 per QALY for an average incidence of 0.00225 per women-year (1.9 years screening interval)	Screening periodicity (continuous time measure) according to breast cancer risk (continuous risk measure)	ICER, cost per QALY
Vilaprinyo 2014	Lee-Zelen probabilistic model (multiestate model)	Women 40–79 years	Mammographic density, family history, previous biopsy	Low: BI-RADS A + one risk factor (RF) among: family history, or previous biopsies, or BI-RADS B without RF Medium-Low: BIRADS A + 2 RF; or BIRADS B + 1 RF; or BIRADS C or D without RF Medium-high: BIRADS B + 2 RF; or BIRADS C or D + 1 RF High: BIRADS C or D + 2 RF	2624 strategies: • Screening start age (40, 45, 50 years) • Periodicity (annual, biennial, triennial, and quinquenial) • Screening stop age (69, 74 years)	Benefits: Number of lives extended, and number of QALY gained. Adverse effects: False-positive results, interval cancers and false-negatives, overdiagnosis, DCIS due to screening Costs: ICER, incremental benefit-harm ratio.
Wu 2013	Markov (microsimulation)	Women ≥ 50 years	BRCA, mammographic density, SNPs, BMI, and age at 1st pregnancy	Deciles of risk according to risk score distribution. Percentile 50–60 as reference.	a) Start age based on age at which the 10-year risk equals 1% of the 10-year risk of the 50th percentile of the risk score at age 50 (29 to 69 years). b) Screening interval (0.4 to 8 years) based on interval cancer rate that equals the threshold of triennial mammography for the 50th percentil of risk score. c) Mammography and MRI, or mammography and US based on the improvement in sensitivity obtained from decreasing the interval cancer rate until the percentile equals the median value of the population with triennial mammography alone.	Number of mammograms, incidence of screen-detected cancer, incidence of interval cancer, proportion of interval cancers among breast cancer cases
Schousboe 2011	Markov (cost-utility model)	Women 40–79 years	Mammographic density, family history, previous biopsy	Risk groups based on cost-effectiveness thresholds ($100,000 and $ 50,000 per QALY), and 10-year age groups (40–49, 50–59, 60–69, 70–79), breast density (BI-RADS), and number of risk factors (family history, previous biopsy).	Periodicity (no screening, annual, biennial, every 3–4 years) Strategy re-evaluation every ten years (40–49, 50–59, 60–69, 70–79)	Cost per QALY gained. Number of women screened in a 10-year period to prevent one breast cancer death.
Ahern 2014	Markov (Monte Carlo microsimulation)	Women 30–90 years with > 25% lifetime breast cancer risk	N.S.	Women with a lifetime breast cancer risk ≥ 25%, vs. women with a lifetime risk ≥ 50% and ≥ 75%.	12 strategies: MRI (annual, biennial), mammography + clinic examination (none, 6 months, 1 year, 2 years), screening stop age (50, 74).	Cost, survival (life years), and QALY ICERs
Pashayan 2011	Probabilistic model	Women 35–79 years	Polygenic risk score (18 loci)	Women aged 47–79 years with 10 years absolute risk ≥ 2.5% vs women 35–79 years with 10 years absolute risk = 2.5%.	Mamography in women 47–79 years (absolute 10-year risk ≥ 2.5%) vs. Mammography age 35–79 years with a 10-year absolute risk = 2.5% based on age + SNPs	Number of women in the target population, number of breast cancers potentitally detectable at screening
Gray 2017	Discrete Event Simulation	Women 50–70 years	Cuzick-Tyrer IBIS risk calculator (phenotype, age at menarche, number of pregnancies, age at first delivery, age at menopause, atypical hyperplasia, lubular carcinoma in sit, BMI) improved with mammographic density.	**Four interventions**: **1)** 3 strata based on 10-year risk: < 3.5%, 3.5%-8%, and >8%; **2)** 3 strata based on 10-year risk terciles: lowest risk, intermediate risk, highest risk; **3)** Masking in women with high mammographic density (Volpara density 3 or 4); **4)** Masking in women with high mammographic density + 3 strata based on intervention 1. Comparison group: Current screening mammmogram every 3 years in women aged 50–70 years, and a no-screening strategy.	Interventions 1 and 2: Mammography every 3, 2, or 1 year for the low, intermediate, and high risk groups, respectively; Intervention 3: Additional US for women with high breast density. If high risk woman (10-year risk >8%) additional MRI instead of US; Intervention 4: triennial, biennial, yearly mammography based on risk group for intervention 1. Additional US If high breast density.	QALY of each strategy, Cost, and ICERs
Van Dyck 2012	Markov (cost-efectiveness)	Women ≥ 50 years	SNPs, breast cancer risk calculator, and risk factors available through the electronic health records system.	High and low risk ^2^	High frequency vs low frequency screening strategy ^3^	Total cost, and QALYs

NS: Not specified; RR: Relative Risk; QALY: Quality-adjusted life years; SNPs: Single Nucleotide Polymorphism; ASSURE (Adapting Breast Cancer Screening Strategy Using Personalised Risk Estimation)

BI-RADS, Breast Imaging Reporting and Data System: A, almost entirely fat; B, scattered fibroglandular density; C, heterogeneously dense; D, extremely dense

^1^ Age is a risk factor in all models, except in the model by Omahony et al, which assumes a constant incidence rate of breast cancer in the group aged 50–70 years

^2^ The study does not specify how the risk groups were stratified

^3^ The study does not specify the high and low frequency strategies.

**Table 2:** Characteristics of randomized controlled trials.

Study ID	Study population ^1^	Risk factors	Comparison groups	Intervention/ Strategy	Primary result
**WISDOM**	Women 40–74 years	Previous biopsies, family history, genetic markers	*Comparison group*: annual mammography screening.	• Low risk (40–49 years and <1.3%): start screening at age 50 years. • Medium risk (50–74 years, or40-49 years with risk≥ 1.3%): biennial mammography. • High risk (extremely dense breasts, risk ≥ 0.75% of ER- tumor): annual mammography. • Very high-risk (carriers of genetic mutations): annual mammography + MRI	• Advanced breast cancer: proportion of tumors diagnosed at stage IIB or higher.
	N≈ 100,000		*Intervention group*: Screening based on breast cancer risk, measured using a model including previous biopsies, family history, and genetic markers.		
**TBST**	Women 44–50 years	Age, breast density	*Comparison group*: annual screening from age 44–45 years. Biennial mammography from age 50 years.	Women 44–50 years: Annual mammography vs. annual/biennial mammography based on breast density.	• Cumulative incidence of T2+/node positive tumors across comparison groups and breast density categories. • Cumulative incidence of interval cancer across comparison groups and breast density categories.
	N≈ 33,200		*Intervention group*: Women 44–45 years with dense breast (BI-RADS C, D) at baseline mammography invited for annual screening. Women 44–45 years with fatty breasts (BI-RADS A, B) invited every 2 years. At age 50 years, all women biennial screening.		
**MyPeBS**	Women 40–74 years N≈ 85,000	Age, breast density, family history, previous biopsies, BMI, genetic markers	*Comparison group*: Standard screening based on the recommendations in each participating country. Intervention group: Stratification in 4 risk groups based on the 5-year risk of breast cancer.	Standard screening vs: • Low risk women (<1%): mammography every 4 years. • Medium risk (1–1.66%): biennial mammography (if high density, US every 2 years) • High risk women (1.67–6%): annual mammography (if high density US every year). • Very high risk women (> = 6%): annual mammography + MRI.	• Incidence rate of breast cancer ≥ stage II across comparison groups

ER: Estrogen Receptor; BMI: Body Mass Index; US: ultrasound; MRI: Magnetic Resonance Imaging

BI-RADS: Breast Imaging Reporting and Data System: A, almost entirely fat; B, scattered fibroglandular density; C, heterogeneously dense; D, extremely dense

^1^ The study population listed is the target for recruitment in both groups

## Results

### Study selection

The database searches for primary studies retrieved 2166 citations, of which 1753 were non-experimental studies, and 413 were clinical trials. After exclusion of duplicates, 1120 non-experimental studies and 413 clinical trials were selected for abstract and title reading. After two independent researchers reviewed the references, 63 studies were considered potentially relevant and were screened in full text. In addition, two studies were included after a manual inspection of their references [17,18].

Finally, 13 studies met the inclusion criteria and were considered in the evidence synthesis [17–29]. Nine out of the 13 studies were mathematical modeling studies [19–27], three were randomized controlled trials [18,28,29], and one was an observational pilot study [17]. Details about study inclusion with reasons for exclusion are described in the flowchart (Fig 1). A list of references of excluded studies is provided in S2 File.

Independently of the study design, all personalized interventions were based on the modification of three factors of the screening process: 1) Age at the start and end of screening, most commonly 40 to 74 years; 2) the frequency of screening, usually annual for high-risk women, and triennial or every 4 years for those with low risk; and 3) the screening modality, with ultrasound (US) proposed for women with high breast density and magnetic resonance imaging (MRI) for women at high risk. Stratification of the target population into risk groups was most commonly based on age, family history of breast cancer, previous biopsies, and breast density. Inclusion of genetic variants to define risk groups is gaining relevance as a key risk factor for stratification.

### Characteristics of the studies according to the study design

#### Mathematical modeling studies

A brief summary of the nine mathematical modeling studies is presented in Table 1 and the extended characteristics in S1 Table. The nine studies were published between 2011 and 2017. Four studies used simulation based on Markov models [19,22,24,26], two were based on probabilistic models [21,25], and one used discrete event simulation [27]. In addition, two studies used adaptations of simulation models previously developed and validated by other teams [20,23]: one used the MISCAN model, based on Monte Carlo microsimulation [20], and the other used one of the models developed by the Cancer Intervention and Surveillance Modeling Network (CISNET) [23].

The age range used to define the risk groups and the screening strategies evaluated varied widely among the studies (Table 1). The most common risk factors employed for stratification of the study population were age, breast density, family history of breast cancer, previous benign breast disease, and polygenic risk profiles based on single nucleotide polymorphisms (SNPs). Other variables used were: phenotype, hormone therapy use, age at first delivery, age at first pregnancy, age at menopause, age at menarche, and body mass index. Two of the studies did not specify the risk factors used to define the risk strata but rather simulated their target population using lifetime risk thresholds obtained from external sources [19,23].

Five studies defined the risk stratification groups based on the absolute risk of developing breast cancer at a specific time horizon, generally 10 years [19–21,26,27]. In addition, two studies proposed risk strata based on age, breast density, family history and previous biopsies [22,25], and another proposed two groups of low and high risk, but did not specify how they were constructed [24].

Personalization strategies varied among the studies included. All studies proposed different screening periodicities according to risk. The periodicities ranged from no screening or 8-year interval screening for lower risk groups, to yearly and 6-monthly screening periodicities for higher risk groups. Six studies proposed strategies that varied the screening age range based on individual risk [19,21–23,25,26], and three studies proposed strategies in which another screening modality replaced or was added to mammography, usually MRI or ultrasound [19,26,27].

The most frequent outcome measures were based on quality-adjusted life years (QALY) gained, absolute costs, costs per QALY gained, and the incremental cost-effectiveness ratio (ICER). In addition, five studies also provided indicators of benefits and adverse effects of screening such as the number of mammograms, women screened, cancers detected, and false-positives, the number and proportion of interval cancers, and the percentage of overdiagnosis [21–23,25,26]. The nine modeling studies showed evidence in favor of personalized screening strategies (S1 File).

#### Randomized controlled trials

A brief summary of the three randomized trials is presented in Table 2 and the extended characteristics in S2 Table. Two out of the three randomized trials, WISDOM and TBST, are currently in the recruitment phase [18,28], while the third, MyPeBS, started in January 2018 and began recruitment in July 2019 [29]. The WISDOM trial presented information from a randomized cohort and from an observational cohort, but both are integrated into the trial [18]. The MyPeBS and WISDOM trials target population aged 40 to 70 years [18,29], while the TBST trial is restricted to women aged 44 to 50 years [28]. The TBST study defines risk groups by age and mammographic density [28]. In contrast, WISDOM and MyPeBS will define risk groups using the Breast Cancer Surveillance Consortium individualized breast cancer risk prediction model [30]. In addition, MyPeBS will also use the Tyrer-Cuzick model risk prediction model for women with more than one first-degree relative with a history of breast cancer [31]. Both trials will include genetic and SNP information to improve calculation of individual risk.

TBST will stratify women in two risk groups based on breast density, and will offer annual screening to women aged 44 to 50 years with dense breasts (BI-RADS 3 and 4), and biennial screening to women with BI-RADS 1 and 2. At age 50 years, women will continue with standard biennial mammography [28]. The WISDOM and MyPeBS trials will combine different techniques and periodicities according to risk profiles [18,29]. Both of them stratify women in four risk groups.

As outcome measures, the TBST trial will assess the incidence of interval cancer in each stratum and will also compare the cumulative incidence of advanced stage tumors (> IIB) across groups and according to mammographic density [28]. The WISDOM and MyPeBS trials will assess the proportion of advanced stage tumors (> IIB) and the reduction in the recall rate and the number of biopsies as the main measures of effectiveness [18,29].

#### Observational study

The only observational study that met the inclusion criteria was a non-randomized open prospective study [17]. The study assessed feasibility, performance, and cost of implementing personalized breast cancer screening in women aged 40 to 49 years based on their individual risk. The study evaluated a single participation of targeted women, stratifying them in three risk groups. The main result variable was the cancer detection rate, although data on costs are also briefly provided.

### Quality assessment and risk of bias

#### Risk of bias in mathematical modeling studies

**Fig 2:**
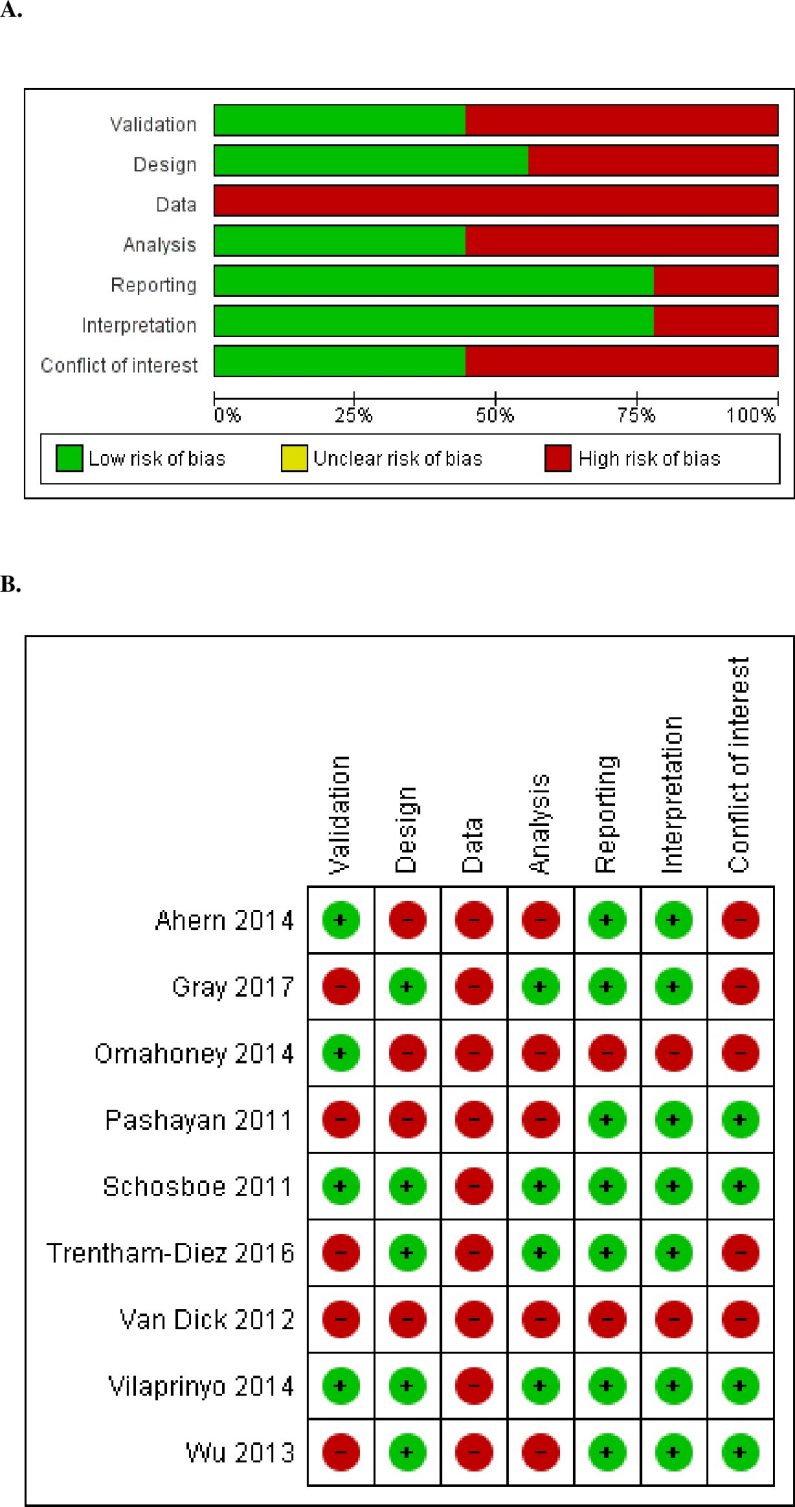
Risk of bias summary: Review of authors' judgments on the risk of bias for mathematical modeling studies by item. **A.** Review authors’ judgments presented as percentage across all mathematical modeling studies. **B.** Risk of bias summary for mathematical modeling studies.

A summary of the risk of bias for mathematical modeling studies is presented in Fig 2, and detailed appraisal and judgments are presented in S3 Table. Overall, the risk of bias in the included studies was moderate due to limitations in the data, validation and model analysis. All studies obtained part of the input data from other studies or external reports. In addition, in one study, the source of part of the input parameters was not specified [20], an in another, the source of information might not be representative of the general population [24]. Four studies did not include validation of the model in the original article [21,23,24,27], while the other five studies included at least internal or external validation [19,20,22,25,26]. Five studies had an appropriate study design based both on the strata dividing the target population and on the evaluated scenarios [22,23,25–27], while one did not stratify women in risk groups [19], one did not specify the risk factor used for stratification [24], one showed several assumptions with potential bias [20], and another used scenarios that were not fully comparable [21].

The outcome measures were considered appropriate and informative. Four studies had a broad set of outcome measures and/or also included extensive additional material describing the assumptions and calculations of their simulations, which endowed high transparency [22,23,25,27]. Reproducibility was assessed as correctly reported in seven studies, while risk of bias was deemed intermediate in one study and high in another. The interpretation was considered balanced in seven studies [19,21–23,25–27] since their main limitations were described and discussed in the discussion, and two other studies had a high risk of bias [20,24]. In addition, five studies did not include a declaration of conflict of interest, three reported having no conflicts of interest, and one reported minimal conflicts of interest that were duly reported.

#### Risk of bias in randomized controlled trials

**Fig 3:**
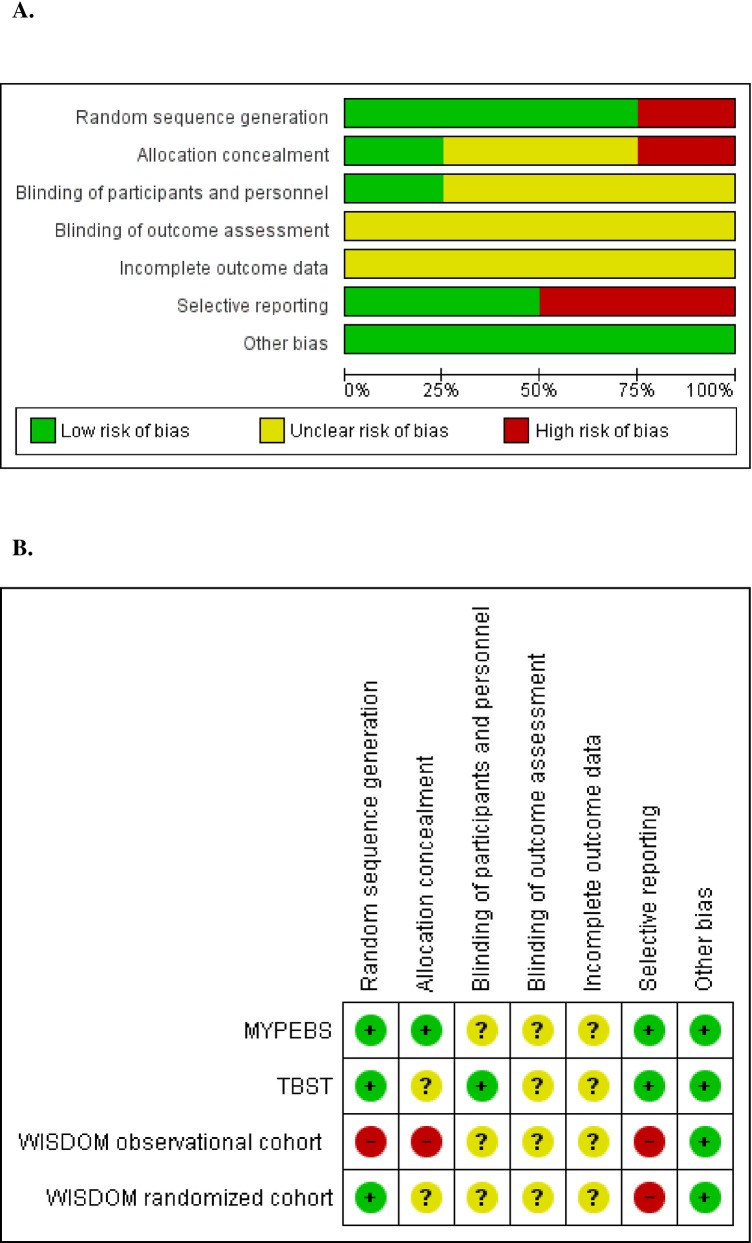
Risk of bias summary: Review of authors’ judgments on the risk of bias of randomized controlled trials by item. **A.** Review authors’ judgments presented as percentages across all randomized controlled trials. **B.** Risk of bias summary for randomized controlled trials.

Overall, the risk of bias was considered low in the trials, despite minor limitations in selection and reporting. A summary of the risk of bias for the randomized trials based on the available protocols is presented in Fig 3, and detailed appraisal and judgments are presented in S4 Table. For the WISDOM trial, we assessed information from the randomized cohort and from the observational cohort separately, since both are integrated into the trial [18].

The random sequence generation was only described in the MyPeBS trial [29]. However, because large studies are based on computer random number generators, the random sequence generation was considered adequate in all three trials, including the WISDOM randomized cohort. In the WISDOM observational cohort, the assignment to an intervention was chosen by participating women, and it was assessed as having a high-risk of bias [18]. The allocation concealment was only consistently described in MyPeBS [29]. In all three studies, the blinding of participants was considered irrelevant. However, the blinding of personnel was not specified in the WISDOM and MyPeBS studies [18,29]. The personnel in TBST was correctly blinded to patient allocation [28]. In all three trials, the main outcome was the incidence of advanced breast cancer, which involves staging of cancers that could be subject to systematic differences between groups in how they are determined. Because of the potential risk of bias, the blinding of outcome was considered unclear in all three trials. In addition, because none of the three studies have published their results yet, we could not assess the bias due to incompleteness of outcome data. TBST and MyPeBS will report outcomes on incidence and cost while the WISDOM trial will not report cost data. Based on the available information, the presence of another type of bias was ruled out.

#### Risk of bias in the observational study

The risk of bias of the observational study was moderate because the study does not include formal validation, neither internal nor external [17]. Its observational pilot design is appropriate. The risk stratification, risk factors, and the scenarios evaluated are correctly identified. The sample size is small, limiting the possible conclusions of the study, but it is considered appropriate to assess feasibility. We also considered the study to be correctly informed, reproducible, and with a balanced interpretation.

## Discussion

### Summary of main results

This systematic review revealed wide heterogeneity in the proposed strategies for personalized breast cancer screening among the 13 included studies. Three studies were randomized controlled trials, nine were mathematical modeling studies, and only one was an observational study.

Despite this heterogeneity, the strategies had a number of common aspects: the population was stratified into risk groups (two to four, mainly), and most strategies included age, family history, previous benign breast disease, and mammographic density as risk factors for stratification. The strategies differed in the screening interval, the age at start or end of screening, and the recommendation of an additional screening test or a substitute for mammography. Screening periodicities according to the risk group varied from annual screening to 8-year intervals or no screening. The start age of screening varied between 35 and 50 years, and the end-of-screening age between 70 and 90 years. Some studies propose MRI or US (in combination or not with mammography) in high-risk women or women with dense breasts (depending on the study).

The most frequent outcome measures in the mathematical modeling studies were QALY, costs, and ICER. However, although there was wide heterogeneity in terms of the strategies evaluated and measures of results, mathematical modeling studies showed that with personalized screening, the gain in QALYs would be higher at a lower cost and with an ICER below the willingness to pay threshold, compared with the standard one-size-fits-all strategy.

In the randomized trials analyzed, the main outcome is the incidence of advanced breast cancer. The rate of advanced cancers is expected to be similar in the first round, when women are randomized, but afterwards, the rate is expected to be higher in the control arm, during the first interval and until the completion of the first round. If the overall cumulative detection rate at the end of the study is similar in the two arms, personalized screening will be more effective at producing early diagnosis. However, if the cumulative detection rate at the end of the study is higher in the experimental arm, it probably means that personalization led to excess overdiagnosis. Both the WISDOM and MyPeBS trials plan to extend the follow-up of study participants to 15 years after study entry to assess the long-term cumulative incidence of breast cancer, the percentage of overdiagnosis, and breast cancer-specific mortality in both arms [18,29].

The main outcome in the observational study was the cancer detection rate. Although presented as a pilot test and with moderate utility, the study showed favorable results for personalization in terms of the detection rate.

### Quality, applicability and completeness of evidence

The quality of the studies was good in the randomized trials. The modeling studies had a moderate risk of bias as a whole, while the observational pilot study had a low risk of bias but its utility was limited to assess the effectiveness of breast cancer screening strategies due to its design and aim.

We found that the three currently ongoing randomized trials are well designed and are likely to provide unbiased results. However, in all three trials, we found a lack of clarity in some important information. For example, in all three trials the outcome is the incidence of advanced breast cancer, which is subject to potential blinding bias, and consequently it was considered unclear. Also, patient allocation, which is key for validation, was clearly reported only in the MyPeBS trial [29]. In addition, because of their experimental nature, trials can only cover a limited number of strategies, and due to their strict inclusion and exclusion criteria, the conclusions may not be valid in a non-controlled real-world scenario. The WISDOM and MyPeBS trials will stratify their target population by means of an individualized breast cancer risk prediction model [18,29]. The TBST trial is targeted to women aged 44 to 50 years and will stratify its target population based on mammographic density alone [28]. Of note, in addition to the classical risk factors, WISDOM and MyPeBS will also collect information through genetic testing, which is expected to improve the discriminatory power of risk predictions to stratify women in risk groups.

There is a need to conduct other randomized controlled trials to assess different strategies and stratification factors to complement the strategies evaluated in the ongoing trials. The evaluation of various radiologic techniques such as tomosynthesis, computer-aided detection, or automated breast US, for instance, is needed to reveal whether personalized strategies are more cost-effective or could lead to a greater number of QALYs. However, such studies are difficult to perform because of factors such as the elevated cost, the length of time to obtain results and probable contamination of the study groups.

Mathematical modeling provides a useful tool to experiment with hypothetical scenarios that are difficult to evaluate in real life. However, their utility is limited and relies on the quality of the input data and the assumptions made to feed the simulation. All the modeling studies in this review obtained all or part of the input data from other studies or external reports, which may affect their external validity. In addition, differences in the distribution of risk factors in the populations included in the models may affect their applicability in other populations. However, despite the uncertainty that they entail, and the heterogeneity in the results measures they use, all the modeling studies reviewed concluded that a personalized screening strategy would be more effective in terms of absolute costs or QALYs than the current uniform strategy within the target population. However, none of the modeling studies assessed mortality reduction or the percentage of overdiagnosis. The results of randomized controlled trials should corroborate some of these findings and consolidate the evidence regarding the greater effectiveness of personalized breast cancer screening.

The evaluation of personalized breast cancer screening strategies through mathematical modeling allows testing of scenarios that would be difficult to assess in real life. Because the models reproduce a system with mathematical concepts, there is a high level of uncertainty in their results. There is also uncertainty in the outcome variables derived directly from the sensitivity of the conceptual model to the input parameters and their mutual interactions. It is important that the conceptual model that interrelates the different parameters is correctly defined, discussed, and agreed upon by a panel of multidisciplinary experts who are aware of the process to be evaluated, in this case breast cancer screening and the natural history of the disease.

In addition, the impact and measurement of some theoretical estimators such as QALYs is open to discussion and varies due to geographic differences and across countries, which could limit the external validity of some studies that include data on cost or utility.

### Potential biases in the review process

It is estimated that the amount of information lost due to the selection criteria of studies is low. An exhaustive literature search was conducted in MEDLINE, EMBASE and the Cochrane Library from its inception. In addition, an exhaustive search of trials in ClinicalTrials.gov and other study databases was conducted in parallel. We did not make an active search of the gray literature, so some studies not indexed in the databases could have been lost, although the probability of this bias is considered very low.

The exclusion criteria used to retrieve the selected studies could be considered too strict. In particular, we excluded studies targeted solely at high-risk populations without an intervention assessed in a comparison group of women with lower or average risk. Given the large number of studies aiming to assess interventions in high-risk women (elevated breast density, BRCA mutation carriers, etc.), this criterion seemed appropriate to narrow down the search to studies addressed at average populations of women targeted for breast cancer screening.

Due to the wide heterogeneity in the approaches, risk factors and stratification criteria assessed in the various studies, as well as the diversity in the outcome variables evaluated, we could not perform a combined analysis of the results of the studies or a meta-analysis. Instead, we presented the search results as a narrative and tabulated synthesis of the characteristics of the studies. In addition, we assessed the risk of bias in each of the studies and the quality of the evidence as a whole.

## Conclusion

As far as we know, this is the first systematic review aiming to identify studies assessing personalized screening strategies as well as to evaluate the quality of the evidence. Few studies have assessed the implementation of personalized breast cancer screening strategies based on women’s individual risk, and their effectiveness has not yet been tested in a real-world population. Mathematical modeling studies, although heterogeneous, showed evidence in favor of personalization based on outcomes such as QALY, costs, and ICER. However, modeling studies do not assess feasibility or acceptance by the target population. Only the observational study showed evidence in favor of personalization in terms of the detection rate, but because of its pilot nature and small scale, it only has moderate utility in adequately assessing specific screening strategies. The three existing randomized controlled trials are still in the recruitment phase and have not reported their results at the time of writing this article. This review shows that there is no conclusive evidence to identify the most advisable personalization strategies. The results suggest the need for additional observational and experimental studies that also assess acceptability, feasibility, and the legal and ethical aspects of personalized screening strategies.

## Supporting information

S1 File Search strategy. (DOCX)

S2 File List of excluded studies with reasons for exclusion. (DOCX)

S1 Table Extended characteristics of mathematical modeling studies. (XLSX)

S2 Table Extended characteristics of randomized controlled trials. (XLSX)

S3 Table Detailed appraisal and judgments of the risk of bias assessment in mathematical modeling studies. (XLSX)

S4 Table Detailed appraisal and judgments of the risk of bias assessment in randomized controlled trials. (XLSX)

## References

[1] Marmot MG, Altman DG, Cameron DA, Dewar JA, Thompson SG, Wilcox M. The benefits and harms of breast cancer screening: an independent review. Br J Cancer. 2013 6 11;108(11):2205–40. 10.1038/bjc.2013.177 23744281 PMC3693450

[2] Perry N, Broeders M, de Wolf C, T”rnberg S, Holland R, von Karsa L. European guidelines for quality assurance in breast cancer screening and diagnosis. 2006; Available from: http://screening.iarc.fr/doc/ND7306954ENC_002.pdf 10.1093/annonc/mdm481 18024988

[3] Bevers TB, Helvie M, Bonaccio E, Calhoun KE, Daly MB, Farrar WB, et al Breast Cancer Screening and Diagnosis, Version 3.2018, NCCN Clinical Practice Guidelines in Oncology. J Natl Compr Cancer Netw JNCCN. 2018 11;16(11):1362–89. 10.6004/jnccn.2018.0083 30442736

[4] Siu AL. Screening for Breast Cancer: U.S. Preventive Services Task Force Recommendation Statement. Ann Intern Med. 2016 2 16;164(4):279–96. 10.7326/M15-2886 26757170

[5] Hofvind S, Sagstad S, Sebuodegard S, Chen Y, Roman M, Lee CI. Interval Breast Cancer Rates and Histopathologic Tumor Characteristics after False-Positive Findings at Mammography in a Population-based Screening Program. Radiology. 2018 4;287(1):58–67. 10.1148/radiol.2017162159 29239711

[6] Hofvind S, Ponti A, Patnick J, Ascunce N, Njor S, Broeders M, et al False-positive results in mammographic screening for breast cancer in Europe: a literature review and survey of service screening programmes. J Med Screen. 2012;19 Suppl 1:57–66. 10.1258/jms.2012.012083 22972811

[7] Roman R, Sala M, Salas D, Ascunce N, Zubizarreta R, Castells X. Effect of protocol-related variables and women’s characteristics on the cumulative false-positive risk in breast cancer screening. Ann Oncol. 2012;23(1):104–11. 10.1093/annonc/mdr032 21430183 PMC3276323

[8] Roman M, Skaane P, Hofvind S. The cumulative risk of false-positive screening results across screening centres in the Norwegian breast cancer screening program. Eur J Radiol. 2014; 10.1016/j.ejrad.2014.05.038 24972452

[9] Rebolj M, Assi V, Brentnall A, Parmar D, Duffy SW. Addition of ultrasound to mammography in the case of dense breast tissue: systematic review and meta-analysis. Br J Cancer. 2018 6;118(12):1559–70. 10.1038/s41416-018-0080-3 29736009 PMC6008336

[10] Evans DG, Kesavan N, Lim Y, Gadde S, Hurley E, Massat NJ, et al MRI breast screening in high-risk women: cancer detection and survival analysis. Breast Cancer Res Treat. 2014 6;145(3):663–72. 10.1007/s10549-014-2931-9 24687378

[11] Berg WA, Zhang Z, Lehrer D, Jong RA, Pisano ED, Barr RG, et al Detection of breast cancer with addition of annual screening ultrasound or a single screening MRI to mammography in women with elevated breast cancer risk. JAMA. 2012 4 4;307(13):1394–404. 10.1001/jama.2012.388 22474203 PMC3891886

[12] Owens DK, Davidson KW, Krist AH, Barry MJ, Cabana M, Caughey AB, et al Risk Assessment, Genetic Counseling, and Genetic Testing for BRCA-Related Cancer: US Preventive Services Task Force Recommendation Statement. JAMA. 2019 8 20;322(7):652–65. 10.1001/jama.2019.10987 31429903

[13] Pashayan N, Morris S, Gilbert FJ, Pharoah PDP. Cost-effectiveness and Benefit-to-Harm Ratio of Risk-Stratified Screening for Breast Cancer: A Life-Table Model. JAMA Oncol. 2018 11 1;4(11):1504–10. 10.1001/jamaoncol.2018.1901 29978189 PMC6230256

[14] Higgins JPT, Green S. Cochrane Handbook for Systematic Reviews of Interventions Version 5.1.0 [Internet]. updated March 2011. The Cochrane Collaboration; 2011 Available from: Available from http://handbook.cochrane.org

[15] Moher D, Liberati A, Tetzlaff J, Altman DG. Preferred reporting items for systematic reviews and meta-analyses: the PRISMA statement. PLoS Med. 2009 7 21;6(7):e1000097 10.1371/journal.pmed.1000097 19621072 PMC2707599

[16] Jaime Caro J, Eddy DM, Kan H, Kaltz C, Patel B, Eldessouki R, et al Questionnaire to assess relevance and credibility of modeling studies for informing health care decision making: an ISPOR-AMCP-NPC Good Practice Task Force report. Value Health J Int Soc Pharmacoeconomics Outcomes Res. 2014 3;17(2):174–82. 10.1016/j.jval.2014.01.003 24636375

[17] Venturini E, Losio C, Panizza P, Rodighiero MG, Fedele I, Tacchini S, et al Tailored breast cancer screening program with microdose mammography, us, and mr imaging: Short-term results of a pilot study in 40-49-year-old wome. Radiology. 2013;268(2):347–55. 10.1148/radiol.13122278 23579052

[18] Esserman LJ. The WISDOM Study: breaking the deadlock in the breast cancer screening debate. NPJ Breast Cancer. 2017;3:34 10.1038/s41523-017-0035-5 28944288 PMC5597574

[19] Ahern CH, Shih YC, Dong W, Parmigiani G, Shen Y. Cost-effectiveness of alternative strategies for integrating MRI into breast cancer screening for women at high risk. Br J Cancer. 2014;111(8):1542–51. 10.1038/bjc.2014.458 25137022 PMC4200098

[20] O’Mahony JF, van RJ, Mushkudiani NA, Goudsmit FW, Eijkemans MJ, Heijnsdijk EA, et al The influence of disease risk on the optimal time interval between screens for the early detection of cancer: a mathematical approach. Med Decis Mak. 2015;35(2):183–95. 10.1177/0272989X14528380 24739535

[21] Pashayan N, Duffy SW, Chowdhury S, Dent T, Burton H, Neal DE, et al Polygenic susceptibility to prostate and breast cancer: implications for personalised screening. Br J Cancer. 2011;104(10):1656–63. 10.1038/bjc.2011.118 21468051 PMC3093360

[22] Schousboe JT, Kerlikowske K, Loh A, Cummings SR. Personalizing mammography by breast density and other risk factors for breast cancer: analysis of health benefits and cost-effectiveness. Ann Intern Med. 2011;155(1):10–20. 10.7326/0003-4819-155-1-201107050-00003 21727289 PMC3759993

[23] Trentham-Dietz A, Kerlikowske K, Stout NK, Miglioretti DL, Schechter CB, Ergun MA, et al Tailoring Breast Cancer Screening Intervals by Breast Density and Risk for Women Aged 50 Years or Older: Collaborative Modeling of Screening Outcomes. Ann Intern Med. 2016;165(10):700–12. 10.7326/M16-0476 27548583 PMC5125086

[24] Van Dyck W, Gassull D, Vértes G, Jain P, Palaniappan M, Schulthess D, et al Unlocking the value of personalised healthcare in Europe—breast cancer stratification. Health Policy Technol. 2012 6 1;1(2):63–8.

[25] Vilaprinyo E, Forne C, Carles M, Sala M, Pla R, Castells X, et al Cost-effectiveness and harm-benefit analyses of risk-based screening strategies for breast cancer. PLoS One. 2014;9(2). 10.1371/journal.pone.0086858 PMC3911927 24498285

[26] Wu YY, Yen MF, Yu CP, Chen HH. Individually tailored screening of breast cancer with genes, tumour phenotypes, clinical attributes, and conventional risk factors. Br J Cancer. 2013;108(11):2241–9. 10.1038/bjc.2013.202 23674086 PMC3681026

[27] Gray E, Donten A, Karssemeijer N, van Gils C, Evans DG, Astley S, et al Evaluation of a Stratified National Breast Screening Program in the United Kingdom: An Early Model-Based Cost-Effectiveness Analysis. Value Health. 2017 9;20(8):1100–9. 10.1016/j.jval.2017.04.012 28964442

[28] TBST study group. Tailored Screening for Breast Cancer in Premenopausal Women (TBST) [Internet]. Bethesda (MD): National Library of Medicine (US): ClinicalTrials.gov; 2015. Report No.: NCT02619123. Available from: https://clinicaltrials.gov/ct2/show/NCT02619123

[29] MyPeBS. Randomized Comparison Of Risk-Stratified versus Standard Breast Cancer Screening In European Women Aged 40–70 (MyPeBS) [Internet]. 2017. Available from: www.brumammo.be/…/bmm-my-pebs-clinical-trial-protocol.pdf

[30] Tice JA, Miglioretti DL, Li C-S, Vachon CM, Gard CC, Kerlikowske K. Breast Density and Benign Breast Disease: Risk Assessment to Identify Women at High Risk of Breast Cancer. J Clin Oncol Off J Am Soc Clin Oncol. 2015 10 1;33(28):3137–43. 10.1200/JCO.2015.60.8869 PMC4582144 26282663

[31] Tyrer J, Duffy SW, Cuzick J. A breast cancer prediction model incorporating familial and personal risk factors. Stat Med. 2004 4 15;23(7):1111–30. 10.1002/sim.1668 15057881

