# Supplementary material for: Personalized breast cancer screening strategies: A systematic review and quality assessment
Source: PLoS One. 2019 Dec 16;14(12):e0226352. doi: 10.1371/journal.pone.0226352 (PMC6913984; doi:10.1371/journal.pone.0226352)
Supplement: S1 File — (DOCX) [file pone.0226352.s001.docx]

**Supporting file 1. Search strategy**

| **MEDLINE**  (Through  PubMed)  **Search date**  January 19^th^, 2018 | #1 Search "Breast Neoplasms"[Mesh] OR  #2 Search breast[Title] AND (neoplasm[Title] OR neoplasms[Title] OR cancer[Title] OR cancers[Title] OR tumor[Title] OR tumors[Title] OR carcinoma[Title/Abstract] OR carcinomas[Title])  #3 Search #1 OR #2  #4 Search ("Mass Screening"[Mesh] OR "Early Detection of Cancer"[Mesh])  #5 Search (screen*[Title] OR "early diagnosis"[Title] OR "early detection"[Title])  #6 Search #4 OR #5  #7 Search (strategy[Title/Abstract] OR strategies[Title/Abstract] OR personalized[Title/Abstract] OR personalised[Title/Abstract] OR individualized[Title/Abstract] OR individualised[Title/Abstract] OR tailored[Title/Abstract] OR risk-based[Title/Abstract])  #9 Search #3 AND #6 AND #7  #10 Search #9 Filters: Publication date from 2010/01/01 703  #11 Search (radiotherap*[Title] OR radiation[Title] OR recurrence[Title] OR recurrences[Title] OR relapse[Title] OR relapses[Title] OR recrudescence[Title] OR recrudescences[Title] OR recurrent[Title] OR metastasis[Title] OR metastases[Title] OR metastat*[Title] OR lymphedema[Title] OR lymphedemas[Title] OR surger*[Title] OR surgic*[Title])  #12 Search #10 NOT #11 685 |
| --- | --- |
| **EMBASE**  (Through EbscoHost)  **Search date**  January 19^th^, 2018 | 1 breast cancer/ or breast tumor/ or breast carcinoma/  2 (breast and (cancer or cancers or neoplasm or neoplasms or tumor or tumors or carcinoma or carcinomas)).ti.  3 1 or 2  4 cancer screening/ or screening/ or mass screening/  5 early cancer diagnosis/  6 screen*.ti.  7 (early adj2 (diagnosis or detection)).ti.  8 4 or 5 or 6 or 7  9 (strategy or strategies or personalized or personalised or individualized or individualised or tailored or risk-based or risk-group).ab,ti. 1209024  10 3 and 8 and 9  11 limit 10 to conference abstracts  13 10 not 11  14 limit 12 to yr="2010 -Current" 1034  15 (radiotherap* or radiation or recurrence or recurrences or recurrent or relaps* or recrudescence or recrudescences or metastasis or metastases or metastat* or lymphedema or lymphedemas or surger* or surgic*).ti.  16 14 not 15 998 |
| **The Cochrane Library**  **Search date**  January 19^th^, 2018 | #1 MeSH descriptor: [Breast Neoplasms] explode all trees  #2 breast:ti and cancer or cancers or neoplasm or neoplasms or tumor or tumors or carcinoma or carcinomas:ti (Word variations have been searched)  #3 #1 or #2 21731  #4 MeSH descriptor: [Mass Screening] explode all trees  #5 MeSH descriptor: [Early Detection of Cancer] explode all trees  #6 screen*:ti or "early diagnosis" or "early detection":ti (Word variations have been searched)  #7 #4 or #5 or #6 12052  #8 strategy or strategies:ti,ab,kw (Word variations have been searched)  #9 personalized or personalised or individualized or individualised or tailored:ti,ab,kw or risk-based or risk-group:ti,ab,kw (Word variations have been searched)  #10 #8 or #9 57324  #11 #3 and #7 and #10 Publication Year from 2010 to 2017 73  #12 radiotherap* or radiation:ti (Word variations have been searched)  #13 recurrence or recurrences or recurrent or relaps* or recrudescence or recrudescences:ti (Word variations have been searched)  #14 metastasis or metastases or metastat*:ti (Word variations have been searched)  #15 lymphedema or lymphedemas:ti,ab,kw (Word variations have been searched)  #16 surger*:ti (Word variations have been searched)  #17 surgic*:ti (Word variations have been searched)  #18 #12 or #13 or #14 or #15 or #16 or #17 85693  #19 #11 not #18 70 |
