# Supplementary material for: Personalized breast cancer screening strategies: A systematic review and quality assessment
Source: PLoS One. 2019 Dec 16;14(12):e0226352. doi: 10.1371/journal.pone.0226352 (PMC6913984; doi:10.1371/journal.pone.0226352)
Supplement: S2 File — (DOCX) [file pone.0226352.s002.docx]

**Supporting file 2. List of excluded studies with reasons for exclusion**

| **Narrative reviews, letters to the editor, editorials, communications to conferences that did not specifically assess personalized screening strategies, N=11** | |
| --- | --- |
| **Study ID** | **References** |
|  | Eby PR. Evidence to Support Screening Women Annually. Radiol Clin North Am. 2017;55(3):441-56. |
|  | Onega T, Beaber EF, Sprague BL, Barlow WE, Haas JS, Tosteson AN, et al. Breast cancer screening in an era of personalized regimens: a conceptual model and National Cancer Institute initiative for risk-based and preference-based approaches at a population level. Cancer. 2014;120(19):2955-64. |
|  | Cancer screening with digital mammography for women at average risk for breast cancer, magnetic resonance imaging (MRI) for women at high risk: an evidence-based analysis. Ont Health Technol Assess Ser. 2010;10(3):1-55. |
|  | Autier P, Boniol M. Mammography screening: A major issue in medicine. European Journal of Cancer. 2018;90(pp 34-62):62. |
|  | Gilbert FJ, Selamoglu A. Personalised screening: Is this the way forward? Clinical Radiology. 2017(no pagination). |
|  | Heller SL, Moy L. Breast MRI Screening: Benefits and Limitations. Current Breast Cancer Reports. 2016;8(4):248-57 |
|  | Burda BU, Norris SL, Holmer HK, Ogden LA, Smith ME. Quality varies across clinical practice guidelines for mammography screening in women aged 40-49 years as assessed by AGREE and AMSTAR instruments. J Clin Epidemiol. 2011;64(9):968-76. |
|  | Bryant H. Of babies and bathwater: Reconsidering the public health approach to breast cancer screening. Cancer Forum. 2014;38(3):187-91. |
|  | Debald M, Wolfgarten M, Walgenbach-Brunagel G, Kuhn W, Braun M. Non-invasive proteomics-thinking about personalized breast cancer screening and treatment. EPMA J. 2010;1(3):413-20. |
|  | Schrager S, Marko K. Mammography at age 40? A risk-based strategy. Journal of Family Practice. 2013;62(11):630-8. |
|  | Duffy SW, Chen THH, Smith RA, Yen AMF, Tabar L. Real and artificial controversies in breast cancer screening. Breast Cancer Management. 2013;2(6):519-28. |
| **Narrative reviews, letters to the editor, editorials, communications to conferences that did not specifically assess personalized screening strategies, N=18** | |
|  | Carles M, Vilaprinyo E, Cots F, Gregori A, Pla R, Roman R, et al. Cost-effectiveness of early detection of breast cancer in Catalonia (Spain). BMC Cancer. 2011;11:192 |
|  | Carter KJ, Castro F, Morcos RN. Insights Into Breast Cancer Screening: A Computer Simulation of Two Contemporary Screening Strategies. AJR Am J Roentgenol. 2018:1-8. |
|  | Huang X, Li Y, Song J, Berry DA. A Bayesian Simulation Model for Breast Cancer Screening, Incidence, Treatment, and Mortality. Med Decis Making. 2017:272989X17714473. |
|  | Zhao Y, Xiong P, McCullough LE, Miller EE, Li H, Huang Y, et al. Comparison of Breast Cancer Risk Predictive Models and Screening Strategies for Chinese Women. J Womens Health (Larchmt). 2017;26(3):294-302. |
|  | Yaffe MJ, Mittmann N, Lee P, Tosteson AN, Trentham-Dietz A, Alagoz O, et al. Clinical outcomes of modelling mammography screening strategies. Health Rep. 2015;26(12):9-15. |
|  | Yaffe MJ, Mittmann N, Lee P, Tosteson AN, Trentham-Dietz A, Alagoz O, et al. Modelling mammography screening for breast cancer in the Canadian context: Modification and testing of a microsimulation model. Health Rep. 2015;26(12):3-8. |
|  | Mandelblatt JS, Stout NK, Schechter CB, van den Broek JJ, Miglioretti DL, Krapcho M, et al. Collaborative Modeling of the Benefits and Harms Associated With Different U.S. Breast Cancer Screening Strategies. Ann Intern Med. 2016;164(4):215-25. |
|  | Mittmann N, Stout NK, Lee P, Tosteson AN, Trentham-Dietz A, Alagoz O, et al. Total cost-effectiveness of mammography screening strategies. Health Rep. 2015;26(12):16-25. |
|  | Gunsoy NB, Garcia-Closas M, Moss SM. Estimating breast cancer mortality reduction and overdiagnosis due to screening for different strategies in the United Kingdom. Br J Cancer. 2014;110(10):2412-9. |
|  | O'Donoghue C, Eklund M, Ozanne EM, Esserman LJ. Aggregate cost of mammography screening in the United States: comparison of current practice and advocated guidelines. Ann Intern Med. 2014;160(3):145. |
|  | Melnikow J, Tancredi DJ, Yang Z, Ritley D, Jiang Y, Slee C, et al. Program-specific cost-effectiveness analysis: breast cancer screening policies for a safety-net program. Value Health. 2013;16(6):932-41. |
|  | Dittus K, Geller B, Weaver DL, Kerlikowske K, Zhu W, Hubbard R, et al. Impact of mammography screening interval on breast cancer diagnosis by menopausal status and BMI. J Gen Intern Med. 2013;28(11):1454-62. |
|  | Duffy SW, Mackay J, Thomas S, Anderson E, Chen TH, Ellis I, et al. Evaluation of mammographic surveillance services in women aged 40-49 years with a moderate family history of breast cancer: a single-arm cohort study. Health Technol Assess. 2013;17(11):vii-95. |
|  | Ahern CH, Cheng Y, Shen Y. Risk-specific optimal cancer screening schedules: an application to breast cancer early detection. StatBiosci. 2011;3(2):169-86. |
|  | Mandelblatt JS, Cronin KA, Berry DA, Chang Y, de Koning HJ, Lee SJ, et al. Modeling the impact of population screening on breast cancer mortality in the United States. Breast. 2011;20 Suppl 3:S75-S81. |
|  | Gotzsche PC, Nielsen M. Screening for breast cancer with mammography. Cochrane Database Syst Rev. 2011(1):CD001877. |
|  | Zelle SG, Vidaurre T, Abugattas JE, Manrique JE, Sarria G, Jeronimo J, et al. Cost-effectiveness analysis of breast cancer control interventions in Peru. PLoS One. 2013;8(12). |
|  | Pataky R, Phillips N, Peacock S, Coldman AJ. Cost-effectiveness of population-based mammography screening strategies by age range and frequency. Journal of Cancer Policy. 2014;2(4):97-102. |
| **Randomized controlled trials not designed to assess personalized screening strategies, N=2** | |
|  | Roberto A, Colombo C, Candiani G, Giordano L, Mantellini P, Paci E, et al. Personalised informed choice on evidence and controversy on mammography screening: Study protocol for a randomized controlled trial. BMC Cancer. 2017;17(1). |
|  | Giordano L, Stefanini V, Senore C, Frigerio A, Castagno R, Marra V, et al. The impact of different communication and organizational strategies on mammography screening uptake in women aged 40-45 years. Eur J Public Health. 2012;22(3):413-8. |
| **Studies applied to high-risk population without a low/intermediate comparison group, N=7** | |
|  | Saadatmand S, Rutgers EJ, Tollenaar RA, Zonderland HM, Ausems MG, Keymeulen KB, et al. Breast density as indicator for the use of mammography or MRI to screen women with familial risk for breast cancer (FaMRIsc): a multicentre randomized controlled trial. BMC Cancer. 2012;12:440. |
|  | Pataky R, Ismail Z, Coldman AJ, Elwood M, Gelmon K, Hedden L, et al. Cost-effectiveness of annual versus biennial screening mammography for women with high mammographic breast density. J Med Screen. 2014;21(4):180-8. |
| 1. ; | de Bock GH, Vermeulen KM, Jansen L, Oosterwijk JC, Siesling S, Dorrius MD, et al. Which screening strategy should be offered to women with BRCA1 or BRCA2 mutations? A simulation of comparative cost-effectiveness. Br J Cancer. 2013;108(8):1579-86. |
|  | Grann VR, Patel PR, Jacobson JS, Warner E, Heitjan DF, Ashby TM, et al. Comparative effectiveness of screening and prevention strategies among BRCA1/2-affected mutation carriers (Structured abstract). Breast cancer research and treatment. 2011;125:837-47. |
|  | Lowry KP, Lee JM, Kong CY, McMahon PM, Gilmore ME, Cott Chubiz JE, et al. Annual screening strategies in BRCA1 and BRCA2 gene mutation carriers: a comparative effectiveness analysis. Cancer. 2012;118(8):2021-30. |
|  | Lee HY, Ju E, Vang PD, Lundquist M. Breast and cervical cancer screening disparity among Asian American women: does race/ethnicity matter [corrected]? J Womens Health (Larchmt). 2010;19(10):1877-84. |
|  | Trop I, Lalonde L, Mayrand MH, David J, Larouche N, Provencher D. Multimodality breast cancer screening in women with a familial or genetic predisposition. Curr Oncol. 2010;17(3):28-36. |
| **Did not propose personalize breast cancer screening strategies, N= 9** | |
|  | Giordano L, Bisanti L, Salamina G, Ancelle PR, Sancho-Garnier H, Espinas J, et al. The EUROMED CANCER network: state-of-art of cancer screening programmes in non-EU Mediterranean countries. Eur J Public Health. 2016;26(1):83-9. |
|  | Neal CH, Rahman WT, Joe AI, Noroozian M, Pinsky RW, Helvie MA. Harms of Restrictive Risk-Based Mammographic Breast Cancer Screening. AJR Am J Roentgenol. 2018;210(1):228-34. |
|  | Winkel RR, Euler-Chelpin MV, Lynge E, Diao P, Lillholm M, Kallenberg M, et al. Risk stratification of women with false-positive test results in mammography screening based on mammographic morphology and density: A case control study. Cancer Epidemiol. 2017;49:53-60. |
|  | Price ER, Keedy AW, Gidwaney R, Sickles EA, Joe BN. The Potential Impact of Risk-Based Screening Mammography in Women 40-49 Years Old. AJR Am J Roentgenol. 2015;205(6):1360-4. |
|  | Sullivan CL, Pandya A, Min RJ, Drotman M, Hentel K. The development and implementation of a patient-centered radiology consultation service: a focus on breast density and additional screening options. Clin Imaging. 2015;39(5):731-4. |
|  | Timmers J, Den HG, Zonderland H, Verbeek A, Broeders M. Changes in the Dutch breast cancer-screening programme. [Dutch]. Huisarts en Wetenschap. 2012;55(7):296-300. |
|  | Evans DG, Warwick J, Astley SM, Stavrinos P, Sahin S, Ingham S, et al. Assessing individual breast cancer risk within the U.K. National Health Service Breast Screening Program: a new paradigm for cancer prevention. Cancer PrevRes (Phila). 2012;5(7):943-51. |
|  | Darabi H, Czene K, Zhao W, Liu J, Hall P, Humphreys K. Breast cancer risk prediction and individualised screening based on common genetic variation and breast density measurement. Breast Cancer Res. 2012;14(1):R25. |
|  | Hellquist BN, Czene K, Hjalm A, Nystrom L, Jonsson H. Effectiveness of population-based service screening with mammography for women ages 40 to 49 years with a high or low risk of breast cancer: Socioeconomic status, parity, and age at birth of first child. Cancer. 2015;121(2):251-8. |
| **Extensions or ampliations of previously published studies included in the review, N= 3** | |
|  | Shieh Y, Eklund M, Madlensky L, Sawyer SD, Thompson CK, Stover FA, et al. Breast Cancer Screening in the Precision Medicine Era: Risk-Based Screening in a Population-Based Trial. J NatlCancer Inst. 2017;109(5). |
|  | Pashayan N, Hall A, Chowdhury S, Dent T, Pharoah PDP, Burton H. Public health genomics and personalized prevention: Lessons from the COGS project. Journal of Internal Medicine. 2013;274(5):451-6. |
|  | Pashayan N, Hall A, Chowdhury S, Dent T, Pharoah PD, Burton H. Public health genomics and personalized prevention: lessons from the COGS project. J Intern Med. 2013;274(5):451-6. |
